# Extramammary Paget's disease of the penis: a case report and review of the literature

**DOI:** 10.1186/1752-1947-3-4

**Published:** 2009-01-06

**Authors:** Kingsley C Ekwueme, Hani D Zakhour, Nigel J Parr

**Affiliations:** 1 Regional Cancer Centre, Department of Urology, Wirral University Teaching Hospital, Arrowe Park Road, Upton, Wirral, CH49 5PE, UK; 2 Department of Histopathology and Clinical Cytology, Wirral University Teaching Hospital, Arrowe Park Road, Upton, Wirral, CH49 5PE, UK

## Abstract

**Introduction:**

Extramammary Paget's disease is a rare cutaneous, slow growing, intraepithelial adenocarcinoma developing in the apocrine gland-bearing areas. Isolated Paget's disease of the penis is extremely rare.

**Case presentation:**

We describe the case of an 87-year-old Caucasian male who presented with a non-healing erythematous plaque on the shaft of the penis previously misdiagnosed as Bowen's disease. The diagnosis was made histologically on the excised specimen and was supported by immunohistochemical staining.

**Conclusion:**

Extramammary Paget's disease is a rare disease which can mimic various types of dermatosis. A high index of suspicion is required, combined with biopsy and immunohistochemical staining in order to make the correct diagnosis. Long-term follow-up is mandatory in these patients in order to identify and treat any subsequent recurrence or concurrent malignancy.

## Introduction

Extamammary Paget's disease (EMPD) is a rare cutaneous, intraepithelial adenocarcinoma involving primarily the epidermis but occasionally extending into the underlying dermis. It has predilection for apocrine gland-bearing areas: mostly the perineum, vulva, axilla, scrotum and penis. Isolated Paget's disease of the penis is rare and only a few cases have been reported in the literature [1].

We describe a case of EMPD confined to the shaft of the penis and initially misdiagnosed on punch biopsy. We also review the literature and highlight the need for a high index of suspicion in the diagnosis of this rare neoplasm.

## Case presentation

An 87-year-old Caucasian male was referred to our centre by a dermatologist, having undergone punch biopsy of a penile lesion with the initial histology reported as showing Bowen's disease. The patient gave a 6-month history of an enlarging lesion on the shaft of his penis prior to presentation to the dermatologist, which had been treated with topical agents and antibiotics. Nevertheless, the dermatologist was clinically suspicious of an invasive lesion prompting referral for wide excision. The patient had had a similar lesion at the same location 10 years earlier which was excised by his general practitioner but no histology report could be traced. He had no other lumps anywhere in the rest of the body and no family history of similar disease. His co-morbidities included ischaemic heart disease, Alzheimer's disease and venous ulcers.

**Figure 1:**
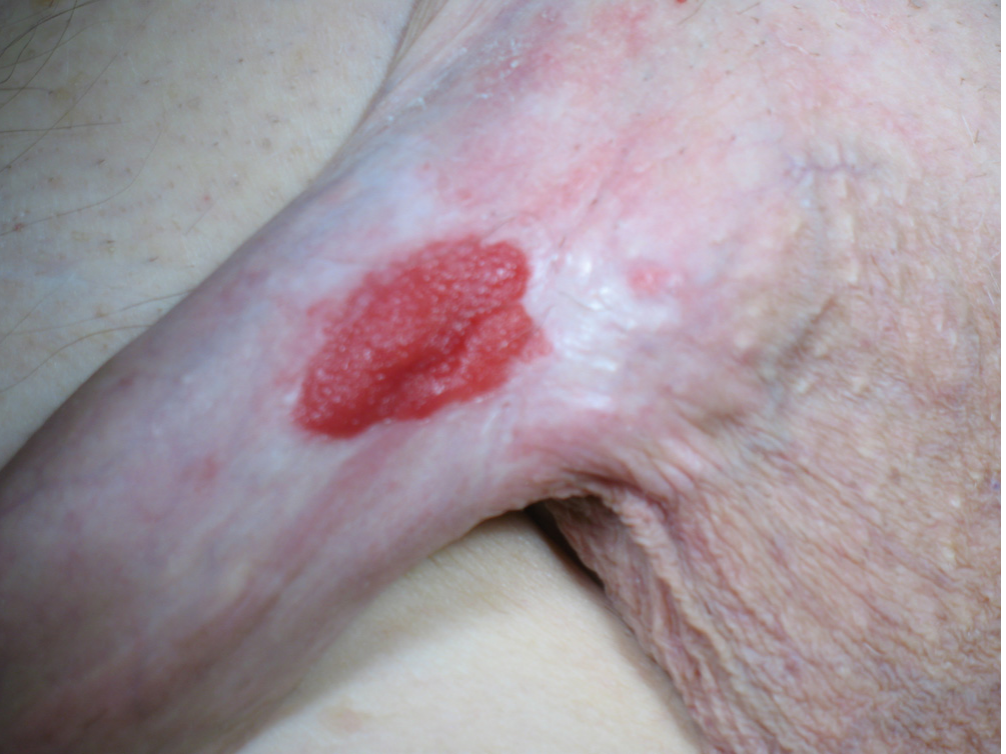
**Photograph of the penile lesion**.

Examination revealed a 2.5 cm erythematous, fleshy, exophytic plaque at the base of the shaft of the penis (Figure 1). There was a satellite lesion proximal to this. The patient had no palpable inguinal lymphadenopathy. A clinical suspicion of an invasive squamous cell carcinoma was made and the patient underwent a wide local excision of the penile and satellite lesions. Frozen-section examination was not performed. The scrotal skin was advanced and primary closure performed. The foreskin was retracted in order to achieve a tension-free closure.

**Figure 2:**
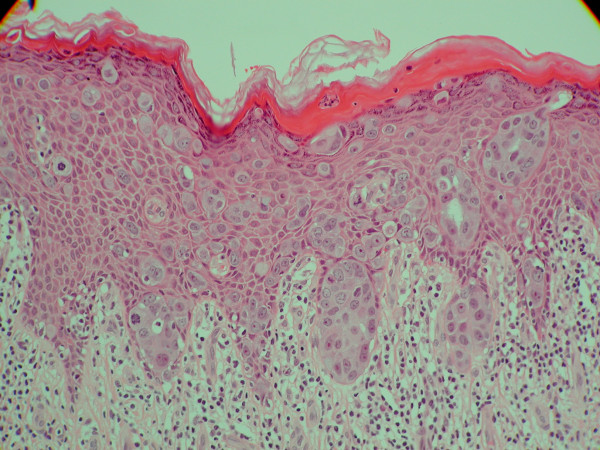
**H&E stain of the resected specimen showing a clear margin**.

**Figure 3:**
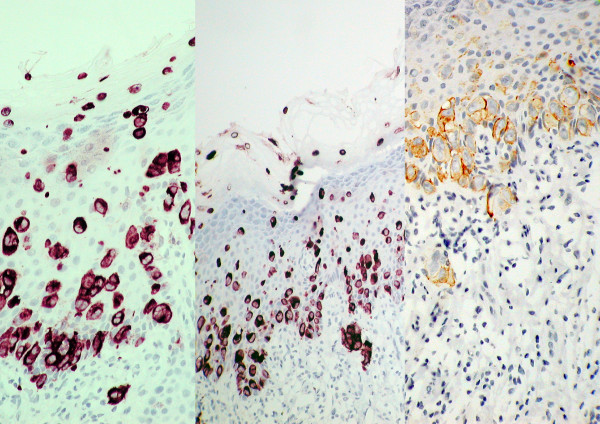
**Immunohistochemical stain of a resected specimen showing occasional cells near the margin**.

The specimen measured 30 × 50 × 50 mm. Light microscopy showed intraepithelial proliferation of neoplastic; large, pale cells, located predominantly in the basal and parabasal layers of the epithelium (Figure 2), with margins apparently clear. Immunohistochemical stains showed specific staining characteristics with strong positivity for epithelial membrane antigen (EMA), the cytokeratin (CK) CK7, CAM 5.6 and HER2 protein over expression. CK20 staining was negative. These immunohistochemical appearances supported the histological diagnosis of EMPD (Figure 3). Immunohistochemical staining also revealed that there were occasional cells in proximity to the margins.

This patient's histology was discussed at our weekly multi-disciplinary cancer meeting and the consensus was not to screen for an underlying non-cutaneous malignancy in view of the patient's age and co-morbidities. Furthermore, a decision was made not to attempt wider excision. At 6-months follow-up, our patient had no local recurrence or palpable inguinal lymph nodes.

## Discussion

EMPD localised to the penis is extremely rare and only few cases have been reported. The first description of EMPD was by Crocker in 1889 when he reported a case affecting the penis and scrotum. EMPD is commoner in females and the elderly population, with a predilection for apocrine gland-bearing areas, most especially the vulva, perianal areas, axilla and penoscrotal region. Other sites reported include the groin, external auditory canal, chest and eyelids.

Clinically, presentation is often non-specific and can mimic any form of dermatosis. Differential diagnoses include Bowen's disease, tinea cruris, contact dermatitis, lichen simplex, lichen planus, psoriasis and seborrheic dermatitis. This can result in delayed presentation as was the case with our patient. In order to make the correct diagnosis, a high index of suspicion is required. The diagnosis is, however, made on histological grounds and supported by immunohistochemical analysis. Positive staining for CK7, a low molecular weight CK, in conjunction with immunonegativity for high molecular weight CKs, have consistently been proven to be the most useful diagnostic markers [2]. This observation was confirmed in our case.

A recent classification based on the origin of the Paget's cells has been proposed by Wilkinson and Brown [3]. They classified vulval Paget's disease (PD) into two broad groups – primary (of cutaneous origin) and secondary (of non-cutaneous origin). For primary PD, Type 1 is primary intraepithelial PD, Type 2 is primary intraepithelial PD with invasion and Type 3 is primary intraepithelial PD as a manifestation of underlying adenocarcinoma of skin appendage origin. Secondary PD originates from an underlying non-cutaneous neoplasm. This proposed classification could help decide on the extent of surgery, prevent unnecessary surgery and influence the outcome.

The true nature of EMPD and its relationship to underlying malignancy remains uncertain. Published reports suggest that up to 42% of patients have associated underlying secondary or non-cutaneous malignancy [4]. However, there is a low incidence of internal malignancy with penoscrotal EMPD [5]. The location of the internal malignancy appears to relate to the location of EMPD. Thus, penoscrotal and perianal locations are associated with adenocarcinoma of the genitourinary and digestive tracts, respectively [6]. Siesling et al. found an increased risk of developing a second primary cancer in their series [7]. Following diagnosis of EMPD, a thorough search for an underlying non-cutaneous malignancy is recommended [6,8]. However, the decision and extent of the search should be tailored to the patient. Chiu et al. [9] recommend screening for only those with perianal or invasive disease and young patients.

The treatment of choice is surgery with wide local excision and immediate reconstruction. Recurrence rates can be up to 60% [9]. Results of frozen section-guided wide, local excision suggest a reduction in the recurrence rate to between 16% and 25% [9,10]. However, the time constraints during surgery mean that assessment of the total margin status by frozen section is difficult and morbidity is likely to increase with prolonged anaesthetic times in frail, elderly patients. In their review, Zhu et al. [10] found a 13% false negative frozen-section analysis. It is unlikely that rates can be reduced further, as positive margins in some cases are only diagnosed by immunohistochemistry. Other treatment modalities which have been used with mixed results include Mohs micrographic surgery, radiotherapy, Nd:YAG and carbon dioxide laser, topical Fluorouracil and 5% imiquimod cream.

The prognosis is good when the disease is confined to the epidermis. However, in the presence of dermal invasion, the prognosis is poor [10].

## Conclusion

PD of the penis is extremely rare. A high index of suspicion, combined with histological examination supported by immunohistochemical staining of biopsy specimen is essential to accurate diagnosis. The treatment of choice is surgery. Frozen section-guided excision reduces the recurrence rate. Long-term follow-up is mandatory in these patients in order to identify and treat any subsequent recurrence or concurrent malignancy.

## Abbreviations

EMPD: extamammary Paget's disease; EMA: epithelial membrane antigen; CK: cytokeratin; PD: Paget's disease

## Consent

Written informed consent was obtained from the patient for publication of this case report and accompanying images. A copy of the written consent is available for review by the Editor-in-Chief of this journal.

## Competing interests

The authors declare that they have no competing interests.

## Authors' contributions

KCE summarized the case and wrote the manuscript. HDZ performed the histological examination of the lesion, reviewed the histology from the referring hospital and provided the histology micrographs, whilst NJP is the Principal Surgeon and provided the overall supervision in the writing of this article. All authors read and approved the final manuscript.
